# Epitome of Diseases and Injuries of the Ear

**Published:** 1888-12

**Authors:** 


*Epitome of Diseases and Injuries of the Ear.* By W. R. H. Stewart, F.R.C.S. Pp. 126. London: H. K. Lewis. 1888.

"Frequently asked by practitioners and students for some work of reference on Ear Diseases small enough to carry in the pocket, the author hopes," &c. This from the preface. Now, what is there peculiar about Ear Diseases, that a book treating of them, "small enough to carry in the pocket," should be so often asked for by practitioners and students? The dainty Elzevir, whether a cookery-book or a classic, had a reason for its diminutive size; but why Ear Diseases? We have nothing to say against the book besides grumbling over its small size and small print, and denouncing the only reason given by the author for writing it—that it should be small enough to carry in the pocket. Some day we shall have tablets of anatomy printed on pocket-handkerchiefs, and dissections modelled in miniature on sleeve-links.

